# Annotations

**Published:** 1908-11-14

**Authors:** 


### Annotations.

### The Plague in England.

As has happened before now in recent years, an isolated case of plague has gained admission into Liverpool, a seaport whose vast shipping operations render it peculiarly exposed to visits from Asiatic disease. The utmost vigilance on the part of the Port Health Authorities cannot prevent an occasional accident of this sort; but early detection, searching inquiries, and the strictest precautions, such as have been brought to bear upon the present case, almost inevitably result in a limitation of the infection. On the evening of Nov. 5, the Local Government Board issued a notice to the effect that a suspicious case of illness had occurred on Oct. 20 in Liverpool, a man engaged in a coaling barge having fallen ill with symptoms which, three days later, were recognised as serious, and ended in his death on Oct. 23. Specimens were sent for bacteriological examination, which on Nov. 3 demonstrated the presence of the bacillus of plague. Meanwhile the fullest precautions had been taken, and all known contacts with this patient were kept under strict supervision. For five days after the man's death nothing further occurred, and it was thought that the exposed persons had escaped infection. Then a man and his wife, who had both been on the barge, and had occupied the cabin before the first patient sought medical advice, were found to be ailing on Nov. 6, and were forthwith isolated in the Port Sanitary Hospital. Symptoms of plague developed in both instances and the man grew rapidly worse on Sunday and died that night. The woman is recovering, and the medical officer reports that no other development has taken place, while the Port Authorities state that no further spread of the plague is to be feared. The primary source of the contagion has not yet been traced, and no diseased rats have been found on the vessel.

### Medical Birthday Honours.

The medical profession has received a very satisfactory share of the honours distributed on Monday last, on the occasion of His Majesty's sixty-seventh birthday. Representatives of widely different branches of the profession from each of the three kingdoms have been rewarded with titles, promotions, or other distinctions; and several prominent workers in connection with hospital administration have also been honoured. Sir George Anderson Critchett, the well-known ophthalmic surgeon, who during the last seven years has held the post of surgeon oculist to the King, receives a baronetcy. A distinction which will be applauded throughout the profession is the Knight Commandership of the Bath which has been granted to the popular and successful President of the General Medical Council. The Universities of Cambridge and Glasgow will feel especial pride in this honour bestowed upon Principal Donald MacAlister. Mr. Jonathan Hutchinson's knighthood, which comes as an appropriate recognition of a long life devoted to the advancement of surgical science and of the interests of the profession, will also cause general satisfaction. Dr. Thomas Oliver, who has also been selected for the honour of knighthood, is Physician to the Royal Victoria Infirmary, Newcastle-on-Tyne, and has rendered notable public service in the investigation and treatment of industrial diseases. In this connection he has served upon several inquiries and congresses, and was for some time medical expert to the Dangerous Trades' Committee of the Home Office. In the list of new knights also appears the name of Dr. Stewart Woodhouse, Physician to the Dublin Children's Hospital, formerly a medical member of the Irish General Prisons Board, and an Inspector under the Local Government Board for Ireland; while in the Royal Victorian Order promotion has been granted to Dr. P. Horton-Smith Hartley, of St. Bartholomew's Hospital, who is honorary secretary to the Executive Committee of the King Edward VII. Sanatorium. Among the recent knighthoods also deserving notice in *The Hospital* are those accorded to Mr. Buxton Morrish and Mr. Frederick Alliston. Both are active workers in the cause of medical charity, and the latter has served for some years as one of the Almoners of St. Bartholomew's Hospital. Lastly, the whole profession will wish to mark its appreciation of the bestowal of the Order of Merit upon the veteran scientist, Dr. Alfred Russel Wallace.

### Applied Bacteriology.

The practical applications of bacteriology in everyday life already include a wide variety of spheres of usefulness, from the septic tank treatment of sewage to the vaccine and serum therapy of human and animal diseases. It would seem, however, that the tremendous latent possibilities of a test-tube containing a culture of bacilli are in some danger of realisation through the ignorance of non-medical traders, who should never have been allowed to handle them. Briefly, a serious outbreak of illness has occurred in a business house in the City which has been traced by the medical officer of health to a much advertised virus, sold for the purpose of destroying rats and mice and alleged to be harmless to human beings and to domesticated animals. Upon removing the flooring of the eating-room of the establishment a large number of dead and decomposing mice were found, a testimonial to the lethal character of the virus, but a proof also that such results may be dearly purchased. There are several of these bacterial preparations on the market, and they are freely exposed for sale by chemists and others without any legal restriction or safeguard. It is claimed for some of these substances that infected animals do not die in their holes, or behind walls, because when approaching death they seek the neighbourhood of water and so die in the open. It is apparent from the evidence that in this particular case such a claim cannot be sustained, and it is also easy to agree with Dr. Collingridge that it is extremely undesirable, and even dangerous, to allow their unfettered distribution. These bacterial vermin-destroyers should be used, if at all, under strict scientific supervision; and the sooner their latent powers of destruction are recognised, the sooner will public opinion arrive at this conclusion.

